# Phenotyping of AMI patients using machine learning: integration of clinical, metabolic, and behavioral features to identify high-risk “frailty–inflammation” phenotype

**DOI:** 10.3389/fcvm.2026.1881280

**Published:** 2026-10-09

**Authors:** Yisha Zhao, Wendi Ding, Hongyi Li, Songqi Guo, Yuying Fan

**Affiliations:** 1 The First Affiliated Hospital of Harbin Medical University, Harbin, Heilongjiang, China; 2 The Second Affiliated Hospital of Harbin Medical University, Harbin, Heilongjiang, China; 3 Department of Nursing, Harbin Medical University, Harbin, Heilongjiang, China

**Keywords:** AMI, frailty-inflammation, hierarchical clustering, machine learning, XGBoost

## Abstract

**Background:**

Prognosis among patients with acute myocardial infarction (AMI) shows substantial heterogeneity, making precise identification of high-risk phenotypes essential for improving long-term outcomes. Machine learning techniques were used to identify such phenotypes by integrating multidimensional data and to examine their association with major adverse cardiovascular events (MACE).

**Methods:**

A total of 1,139 patients with AMI were enrolled from the First Affiliated Hospital of Harbin Medical University between March 2023 and June 2024. Multidimensional data, including clinical features, metabolic characteristics, and behavioral patterns, were collected. Phenotyping was performed using a hierarchical clustering algorithm. Associations between derived phenotypes and 1-year MACE were evaluated using Kaplan–Meier survival analysis and multivariable Cox regression models. Shapley Additive Explanations (SHAP) values derived from an XGBoost model were used to quantify the contributions of individual variables to cluster assignment.

**Results:**

Hierarchical clustering identified two clinical phenotypes with differing pathophysiological characteristics. Cluster 2 (*n* = 243, 21.3%) exhibited a high-risk “frailty–inflammation” phenotype, characterized by impaired activities of daily living, elevated risk of falls and pressure ulcers, increased high-sensitivity C-reactive protein (hs-CRP) levels, and poor health behaviors. Compared with Cluster 1, patients in Cluster 2 showed a higher hazard of 1-year MACE (unadjusted HR = 4.17; 95% CI: 3.237–5.371; *P* < 0.001) and a markedly increased risk of all-cause mortality (HR = 12.162; 95% CI: 7.445–19.868; *P* < 0.001). After adjustment using the minimal sufficient confounding model (M3, incorporating 17 prespecified confounders), Cluster 2 membership remained significantly associated with an increased risk of 1-year MACE (adjusted HR = 2.726; 95% CI 1.986–3.743; *P* < 0.001) and all-cause mortality (adjusted HR = 4.35; 95% CI 2.393–7.905; *P* < 0.001). In multivariable Cox regression analysis, Cluster 2 membership was identified as an independent predictor for MACE.

**Conclusions:**

Integration of multidimensional data using machine learning identifies a high-risk “frailty–inflammation” phenotype in patients with AMI. This phenotype offers a comprehensive biological–functional–behavioral framework for risk stratification and may support early and precise clinical interventions to reduce long-term adverse outcomes. However, despite its potential clinical value, the current evidence is insufficient to support direct translation into routine clinical practice until independent cohort validation is performed. Future multi-center studies are warranted to confirm the robustness and generalizability of this approach, and to prospectively evaluate whether phenotype-guided intervention strategies can improve long-term outcomes.

## 1 Introduction

Acute myocardial infarction (AMI) remains a major global public health challenge, characterized by high incidence, disability, and mortality rates. According to the Global Burden of Disease Study, by 2019, ischemic heart disease, including AMI, accounted for 182 million disability-adjusted life years (DALYs), making it the leading cause of death worldwide (1). Major adverse cardiovascular events (MACE) serve as a key composite endpoint for evaluating treatment effectiveness and clinical prognosis in cardiovascular disease (CVD), typically including all-cause mortality, new-onset heart failure, recurrent myocardial infarction, refractory angina, unplanned coronary revascularization, and stroke (2). Although advances in percutaneous coronary intervention (PCI) have reduced in-hospital mortality among patients with AMI, improvements in long-term prognosis remain limited. A retrospective cohort study in a multi-ethnic Asian population reports that the incidence of MACE within one year after AMI is as high as 14.3% (3). These findings highlight the substantial social and familial burden of AMI and underscore the need to identify high-risk phenotypes to enable precise prognostic stratification.

The AMI population exhibits substantial heterogeneity, which is a key determinant of long-term outcomes (4). Identification of clinical subtypes with shared underlying characteristics is therefore essential for optimizing risk stratification and guiding individualized treatment strategies. Previous clustering studies in AMI primarily rely on traditional statistical approaches. Even when machine learning techniques, such as K-means and hierarchical clustering, are applied (5, 6), analyses are often limited to baseline characteristics, laboratory parameters, and procedural variables. Important aspects of patient status, particularly physical functional status (e.g., frailty) and health-related behaviors (e.g., lifestyle factors associated with inflammation), are frequently overlooked (7, 8). These underexplored variables may represent critical determinants of outcome heterogeneity and may facilitate identification of high-risk phenotypes, including the “frailty–inflammation” phenotype.

Machine learning, a core component of artificial intelligence, enables analysis of high-dimensional data and identification of complex non-linear relationships and latent patterns, thereby capturing information that may be missed by conventional approaches (9). Hierarchical agglomerative clustering (HAC), an unsupervised learning method, constructs clusters through iterative bottom-up merging of similar observations. Its ability to operate without pre-specifying the number of clusters makes it particularly suitable for exploring disease heterogeneity. This approach provides a robust framework for integrating multidimensional data, including clinical, metabolic, and behavioral features, and has strong potential for risk stratification and prognostic assessment in precision medicine (10).

Therefore, this study aims to overcome the limitations of previous work by integrating multidimensional data from the full spectrum of patients with AMI—including clinical indicators, biomarkers, physical functional status, and health behaviors—and applying hierarchical agglomerative clustering for exploratory analysis. Unlike prior studies that have focused on specific infarction types or relied on narrowly defined variable sets, this approach leverages the breadth of multidimensional information across the entire AMI population to better capture the complexity of patient heterogeneity. The objectives are as follows: (1) to identify patient subtypes with similar characteristics using machine learning–based clustering, with emphasis on detecting high-risk phenotypes associated with frailty–inflammation; (2) to compare clinical features across subtypes and perform model-based feature-importance analysis to identify key distinguishing factors; and (3) to evaluate associations between subtypes, particularly the high-risk frailty–inflammation phenotype, and clinical outcomes, thereby providing a foundation for more targeted management strategies across risk strata.

## 2 Methods

### 2.1 Study design

This was an observational cohort study conducted in accordance with the Strengthening the Reporting of Observational Studies in Epidemiology (STROBE) guidelines. An unsupervised machine learning approach was applied to perform data-driven phenotyping of patients with AMI. The primary objectives were to identify distinct patient subtypes characterized by similar clinical features, metabolic characteristics, and behavioral patterns and to evaluate their associations with 1-year clinical outcomes. The study protocol was reviewed and approved by the Ethics Committee of the affiliated institution.

### 2.2 Data source

Consecutive patients admitted to the First Affiliated Hospital of Harbin Medical University between March 1, 2023, and June 28, 2024, with a diagnosis of AMI were initially screened. The diagnosis of AMI was established in accordance with the Fourth Universal Definition of Myocardial Infarction, defined by evidence of acute myocardial injury (elevation of cardiac troponin values with at least one measurement above the 99th percentile upper reference limit) together with clinical evidence of acute myocardial ischemia.

The inclusion criteria were: (1) age ≥ 18 years; (2) fulfillment of AMI diagnostic criteria; and (3) admission via the emergency department. The exclusion criteria were: (1) critical data missing, defined as absence of >20% of core variables used for cluster analysis; (2) non-index admission, defined as hospitalization for complications after the initial AMI or routine follow-up; (3) in-hospital death, defined as death occurring before completion of baseline data collection and physical status assessment; (4) comorbidities with poor prognosis, including end-stage renal disease, severe hepatic failure, active malignancy, or other non-cardiovascular diseases with a life expectancy <1 year; and (5) loss to follow-up, defined as inability to obtain 1-year outcome data via telephone follow-up or electronic health record review. After applying these criteria, 1,139 patients were included in the final analysis.

### 2.3 Data collection and outcome definition

Data collection encompassed multiple domains: (1) **Sociodemographic data:** gender, age, marital status, educational attainment, and occupation; (2) **Clinical characteristics and comorbidities:** admission parameters included heart rate, blood pressure, body mass index (BMI), and Killip class. Medical records were reviewed to obtain smoking status, alcohol consumption, and history of myocardial infarction, hypertension, diabetes, heart failure, stroke, and other cardiac diseases; (3) **Treatment strategies:** in-hospital treatments included percutaneous coronary intervention, coronary artery bypass grafting, thrombolysis, and guideline-recommended secondary prevention medications; (4) **Laboratory and cardiac function parameters:** laboratory analyses were conducted in the hospital central laboratory and included complete blood count, liver and renal function, cardiac enzymes, lipid profile, glucose, B-type natriuretic peptide (BNP), and high-sensitivity C-reactive protein (hs-CRP). Left ventricular ejection fraction (LVEF) was assessed using transthoracic echocardiography; (5) **Physical function assessment:** after clinical stabilization, trained nurses performed standardized assessments using the Modified Barthel Index (activities of daily living; scores 0–100, higher scores indicate greater independence) (11), Braden Scale (pressure ulcer risk; scores 6–23, lower scores indicate higher risk) (12), Morse Fall Scale (fall risk; scores 0–125, higher scores indicate higher risk) (13), and Padua Scale (venous thromboembolism risk; score ≥4 indicates high risk) (14); and (6) **Post-discharge health behaviors:** during the 1-year follow-up, adherence to dietary control, physical activity, medication use, and scheduled follow-up visits was assessed using a structured questionnaire.

The primary outcome was the occurrence of MACE within 1 year after discharge, defined as a composite of: (1) all-cause mortality; (2) recurrent non-fatal myocardial infarction; (3) rehospitalization for refractory angina or new-onset heart failure; and (4) ischemic or hemorrhagic stroke. Endpoint events were independently adjudicated by two cardiologists blinded to cluster assignments.

### 2.4 Statistical analysis

All statistical analyses were performed using R software (version 4.4.0).

Missing data were handled using a stratified, method-adapted strategy: for 39 routine laboratory parameters with low missing rates, mean imputation was applied; for clinically important variables with higher missing rates (including LVEF, cTnI, TnT, NT-proBNP, BNP, hs-CRP, PCT, Gensini score, thyroid function indices, and selected behavioral variables), multiple imputation by chained equations was performed using the mice package (v3.19.0), with m = 5 imputations, maxit = 10 iterations, and a fixed random seed (seed = 123) to ensure reproducibility.

Unsupervised hierarchical agglomerative clustering (HAC) was subsequently performed to identify patient phenotypes based on clinical, metabolic, and behavioral data (Supplementary Table S3). The Euclidean distance metric and Ward.D2 linkage method were used. Prior to clustering, categorical variables were one-hot encoded and continuous variables were Z-score standardized. The optimal number of clusters was determined by integrating multiple validation approaches, including majority voting across 17 successful indices from the NbClust package (v3.0.1), average silhouette width from the cluster package (v2.1.8.3), within-cluster consistency from ConsensusClusterPlus (v1.70.0), and bootstrap stability assessment using clusterboot (B = 500) from the fpc package (v2.2–14). Cluster stability was further evaluated by comparing dendrograms across alternative linkage methods (complete, average, and single).

Categorical variables are presented as frequencies and percentages. Continuous variables were assessed for normality using a comprehensive approach combining the Shapiro–Wilk test, skewness, outlier inspection, and Q-Q plots. Accordingly, variables consistent with a normal distribution were expressed as mean ± standard deviation and compared using the t-test or one-way ANOVA; non-normally distributed variables were expressed as median (Q1, Q3) and compared using the Mann–Whitney U test or Kruskal–Wallis test. Between-cluster comparisons of categorical variables were performed using the chi-square test or Fisher exact test as appropriate (Supplementary Table S1).

Time-to-event analysis for MACE was conducted using the Kaplan–Meier method, with comparisons performed using the log-rank test and numbers at risk reported beneath each curve. Survival analyses were performed using the survival (v3.5–8) and survminer (v0.5.2) packages. Multivariable Cox proportional hazards models were used to estimate associations between cluster membership and 1-year MACE and all-cause mortality, reported as hazard ratios (HRs) with 95% confidence intervals (CIs). Covariates were prespecified based on a directed acyclic graph (DAG) constructed using the dagitty package (v0.3–4) (Supplementary Figure S7), without data-driven selection. The minimal sufficient confounding model (M3) included 17 prespecified confounders: age, sex, marital status, education, occupation, symptom onset-to-admission time, myocardial infarction episodes, renal failure, cardiopathy, DBP, BMI, cTnI, NT-proBNP, WBC, Gensini score, drinking, and sleep (Supplementary Table S6). Model comparison followed a three-tier nested strategy: unadjusted model (M1), demographic-adjusted model (M2), and minimal sufficient confounding model (M3). The proportional hazards (PH) assumption was assessed using Schoenfeld residuals (Supplementary Table S8; Figure S8). For models violating the PH assumption, a cluster×log(time) interaction term was included to formally model time-varying effects, with time-averaged HRs and time-varying HR curves reported (Supplementary Table S10; Figure S9). Sample size adequacy was informed by events-per-variable (EPV) criteria.

To assess the incremental prognostic value of cluster membership beyond established anatomical severity, time-dependent ROC analyses were conducted using the pROC package (v1.19.0.1) and the survIDINRI package (v1.1–2), and decision curve analysis (DCA) was performed using the dcurves package (v0.5.1), comparing the cluster-based model, the Gensini score model, and a combined model. DeLong tests were used to compare AUCs between models. Calibration of the main Cox model was evaluated using 5-fold cross-validated out-of-fold predicted probabilities, with calibration slope, intercept, and Brier score reported. Clinical utility was assessed using DCA across threshold probabilities of 1%–60%, comparing net benefit against “treat all” and “treat none” strategies. It should be noted that GRACE and TIMI risk scores could not be reliably calculated because several required variables were not collected; the Gensini score was therefore used as the reference comparator. Stratified Cox regression by AMI type (STEMI vs. NSTEMI) was performed to evaluate whether the prognostic association of cluster membership differed by infarction type, with interaction *P* values reported.

Discriminative performance of the clustering solution for 1-year outcomes was evaluated using the area under the receiver operating characteristic curve (AUC), calculated with the pROC package (v1.19.0.1). To further explore feature importance, an extreme gradient boosting (XGBoost) model was developed separately for each cluster using the xgboost package (v3.2.1.1), with the same input feature set and no independent feature selection. Hyperparameters were optimized using 5-fold cross-validation (Supplementary Table S13). SHapley Additive exPlanations (SHAP) values were calculated using the SHAPforxgboost package (v0.2.0) to quantify feature contributions, visualized using summary bar plots and beeswarm plots. Additional supporting analyses were performed using the car package (v3.1–5) and the MASS package (v7.3–60.2). A two-sided *P* < 0.05 was considered statistically significant.

## 3 Result

### 3.1 Clustering analysis results and cluster characteristics

A total of 1,139 patients with AMI were included in the final analysis. Hierarchical agglomerative clustering was performed using the Euclidean distance metric and Ward.D2 linkage method (Supplementary Figures S2–4). The optimal number of clusters was determined by integrating multiple validation approaches. Among 24 indices evaluated by the NbClust package, 17 yielded successful results, of which 7 voted for the two-cluster solution (Figure 1A). The silhouette coefficient and consensus clustering results provided consistent support for this choice (Supplementary Figures S1, S5A–B). The cohort was therefore classified into Cluster 1 (*n* = 896, 78.7%) and Cluster 2 (*n* = 243, 21.3%). Baseline characteristics of the two clusters are presented in Supplementary Table S2.

**Figure 1:**
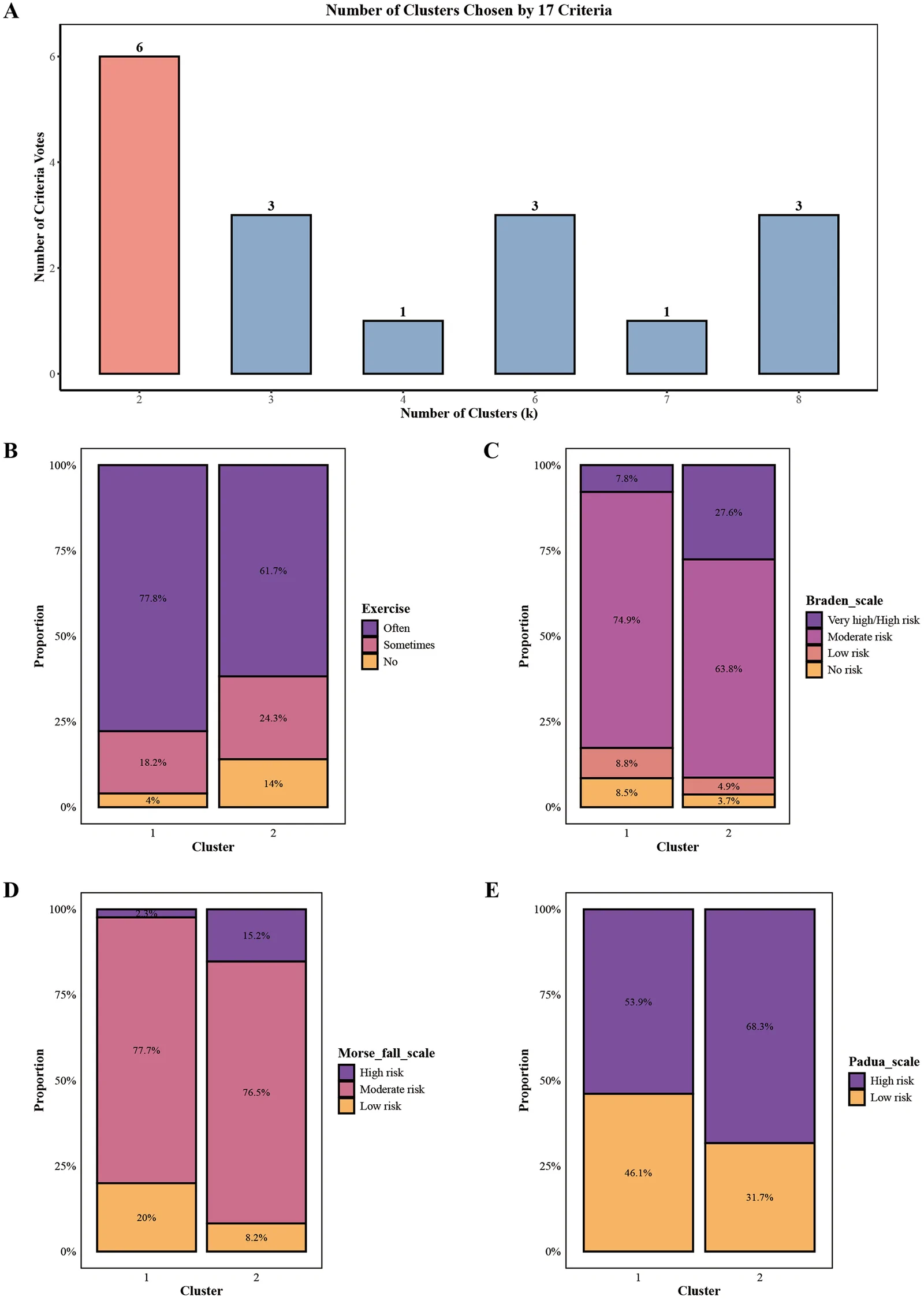
Selection of the optimal number of clusters. **(A)** Evaluation of the optimal number of HAC clusters using 17 indices implemented in the NbClust package. **(B)** Stacked bar chart showing the distribution of exercise categories in Cluster 1 and Cluster 2. **(C)** Stacked bar chart showing the distribution of Braden Scale categories in Cluster 1 and Cluster 2. **(D)** Stacked bar chart showing the distribution of Morse Fall Scale categories in Cluster 1 and Cluster 2. **(E)** Stacked bar chart showing the distribution of Padua Scale categories in Cluster 1 and Cluster 2.

The two clusters differed across multiple domains. Statistically significant differences were observed in all sociodemographic variables, with pronounced differences in sex, age, education, and occupation (all *P* < 0.001). Differences were also observed in most clinical characteristics, particularly Killip class, myocardial infarction episodes, LVEF, and Gensini score (all *P* < 0.001). All metabolic variables differed between clusters, including total cholesterol, triglycerides, low-density lipoprotein cholesterol (all *P* < 0.001), and glucose (*P* = 0.001). The distribution of AMI type (STEMI vs. NSTEMI) also differed significantly between clusters (*P* = 0.006). In addition, most behavioral variables differed, with notable differences in smoking status, physical activity, and follow-up adherence (all *P* < 0.001). Further variables showing differences between clusters included diabetes, renal failure, stroke, cardiac disease, heart rate, systolic and diastolic blood pressure, BMI, Morse Fall Scale, Braden Scale, Padua Scale, NT-proBNP, BNP, hs-CRP, procalcitonin (PLT), hemoglobin (HGB), D-dimer (D-D.P), creatinine, albumin (ALB) and treatment strategies (all *P* < 0.001) (Supplementary Table S2).

Marked differences were also observed in physical function and behavioral variables. To illustrate these patterns, stacked bar charts were constructed to show the distribution of physical activity, Braden Scale, Morse Fall Scale, and Padua Scale across clusters (Figures 1B–E). The proportion of patients engaging in regular physical activity (>10 h/week) was higher in Cluster 1, whereas the proportions of patients classified as high risk according to the Braden Scale, Morse Fall Scale, and Padua Scale were higher in Cluster 2.

### 3.2 Prognostic differences and risk associations of the identified clusters

Kaplan–Meier curves showed that, except for refractory angina (*P* = 0.089) and stroke (*P* = 0.999), Cluster 2 had significantly higher cumulative incidences of major adverse cardiovascular events (MACE), all-cause mortality, recurrent myocardial infarction, and new-onset heart failure compared with Cluster 1 (all *P* < 0.001) (Figures 2A–F).

**Figure 2:**
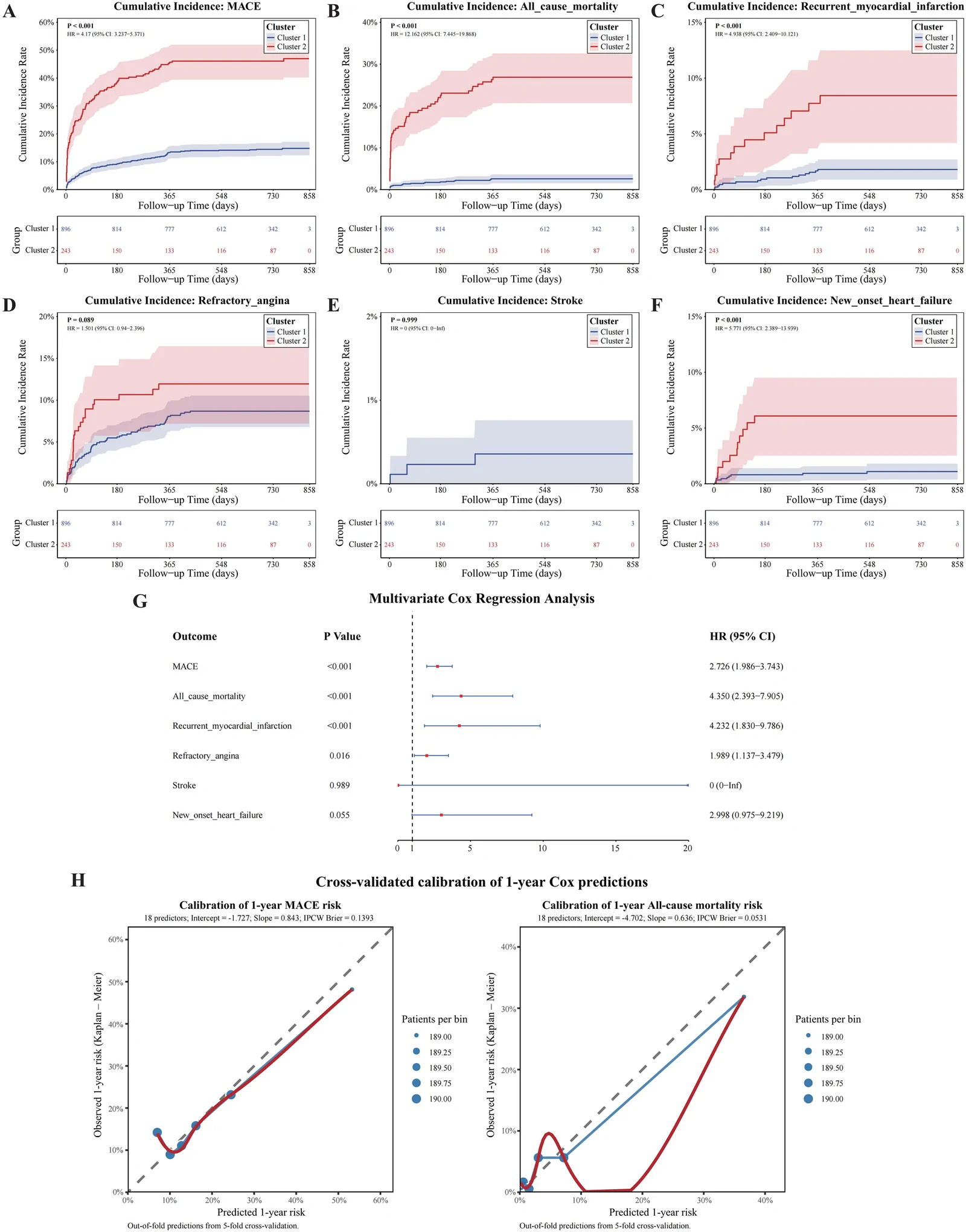
Survival analysis across clusters. **(A)** Kaplan–Meier analysis of MACE in the two clusters. **(B)** Kaplan–Meier analysis of all-cause mortality. **(C)** Kaplan–Meier analysis of recurrent MI. **(D)** Kaplan–Meier analysis of refractory angina. **(E)** Kaplan–Meier analysis of stroke. **(F)** Kaplan–Meier analysis of new-onset HF. **(G)** Forest plot of cluster-associated risks based on multivariable Cox regression. **(H)** Calibration plot for the predicted 1-year risk of MACE (left) and all-cause mortality (right). MACE, major adverse cardiovascular events; MI, myocardial infarction; HF, heart failure.

In the three-tier nested Cox models, Cluster 2 membership was associated with a markedly elevated risk of 1-year MACE in the unadjusted model (M1: HR = 4.17, 95% CI 3.237–5.371, *P* < 0.001). The association remained significant after demographic adjustment (M2: HR = 3.42, 95% CI 2.600–4.502, *P* < 0.001) and after adjustment using the minimal sufficient confounding model (M3, 17 prespecified confounders; adjusted HR = 2.726, 95% CI 1.986–3.743, *P* < 0.001) (Supplementary Table S7). The consistent significance across all three models indicated that the association was not sensitive to adjustment strategy. The attenuation from the unadjusted to the adjusted estimate was probably driven by the older age, higher proportion of female patients, greater comorbidity burden, and worse functional status in Cluster 2 (Supplementary Table S2); nevertheless, the association remained robust after full adjustment. For individual MACE components, Cluster 2 was associated with elevated risks of all-cause mortality (adjusted HR = 4.350, 95% CI 2.393–7.905, *P* < 0.001), recurrent myocardial infarction (adjusted HR = 4.232, 95% CI 1.830–9.786, *P* < 0.001), refractory angina (adjusted HR = 1.989, 95% CI 1.137–3.479, *P* = 0.016), and new-onset heart failure (adjusted HR = 2.998, 95% CI 0.975–9.219, *P* = 0.055). No significant between-cluster difference was observed for stroke (adjusted HR = 0, 95% CI 0–Inf, *P* = 0.989) (Figure 2G).

To evaluate the calibration of the main Cox model, we used 5-fold cross-validated out-of-fold predicted probabilities. For MACE, the calibration slope was 0.843 (95% CI 0.703–0.983), the calibration intercept was −1.727, and the IPCW Brier score was 0.139. For all-cause mortality, the calibration slope was 0.636 (95% CI 0.533–0.740), the intercept was −4.702, and the Brier score was 0.053 (Figure 2H; Supplementary Table S9). Calibration slopes below 1 indicated a tendency toward overestimation of absolute risk in high-risk subgroups, which was more pronounced for all-cause mortality, consistent with the lower number of events and EPV of approximately 4.5 for this endpoint. However, this miscalibration does not affect the comparison of relative risks (HRs) between clusters.

To assess whether the cluster-based classification provides incremental prognostic value beyond established anatomical severity, we compared its performance with the Gensini score. Decision curve analysis demonstrated that the cluster-based model achieved greater net benefit than the “treat all” and “treat none” strategies at threshold probabilities above approximately 12%, and outperformed the Gensini score alone within the 20%–40% threshold range (Supplementary Figures S10A and C). For discriminative performance, the combined model (cluster + Gensini) showed a higher AUC than the Gensini score alone for MACE (0.697 vs. 0.674; DeLong *P* = 0.024) and for all-cause mortality (0.856 vs. 0.838; DeLong *P* = 0.060), although the difference for all-cause mortality did not reach statistical significance (Supplementary Figures S10B and D; Tables S11 and 12). In Gensini-stratified analysis, Cluster 2 remained significantly associated with elevated MACE risk within both the intermediate-risk stratum (HR = 3.852, 95% CI 2.109–7.035) and the high-risk stratum (HR = 2.593, 95% CI 1.497–4.491), with a non-significant interaction (*P* = 0.068) (Supplementary Tables S13 and S14), indicating that the phenotype classification provides additional prognostic information beyond coronary anatomical severity.

Stratified Cox regression by AMI type was performed to evaluate whether the prognostic association of cluster membership differed between STEMI and NSTEMI. The effect of Cluster 2 on 1-year MACE was directionally consistent across both AMI types, with a non-significant interaction (Supplementary Table S15; Figure S11), indicating that the “frailty–inflammation” high-risk phenotype and its prognostic association are applicable to both STEMI and NSTEMI patients.

### 3.3 Prognostic performance evaluation and SHAP visualization

To evaluate the discriminative ability of the cluster-based classification for 1-year MACE and all-cause mortality, receiver operating characteristic (ROC) curves were constructed. The AUC was 0.708 for MACE prediction and 0.811 for all-cause mortality prediction (Figures 3A,B), indicating that the two-cluster solution itself possessed moderate to good discriminative capacity for distinguishing outcome event status.

**Figure 3:**
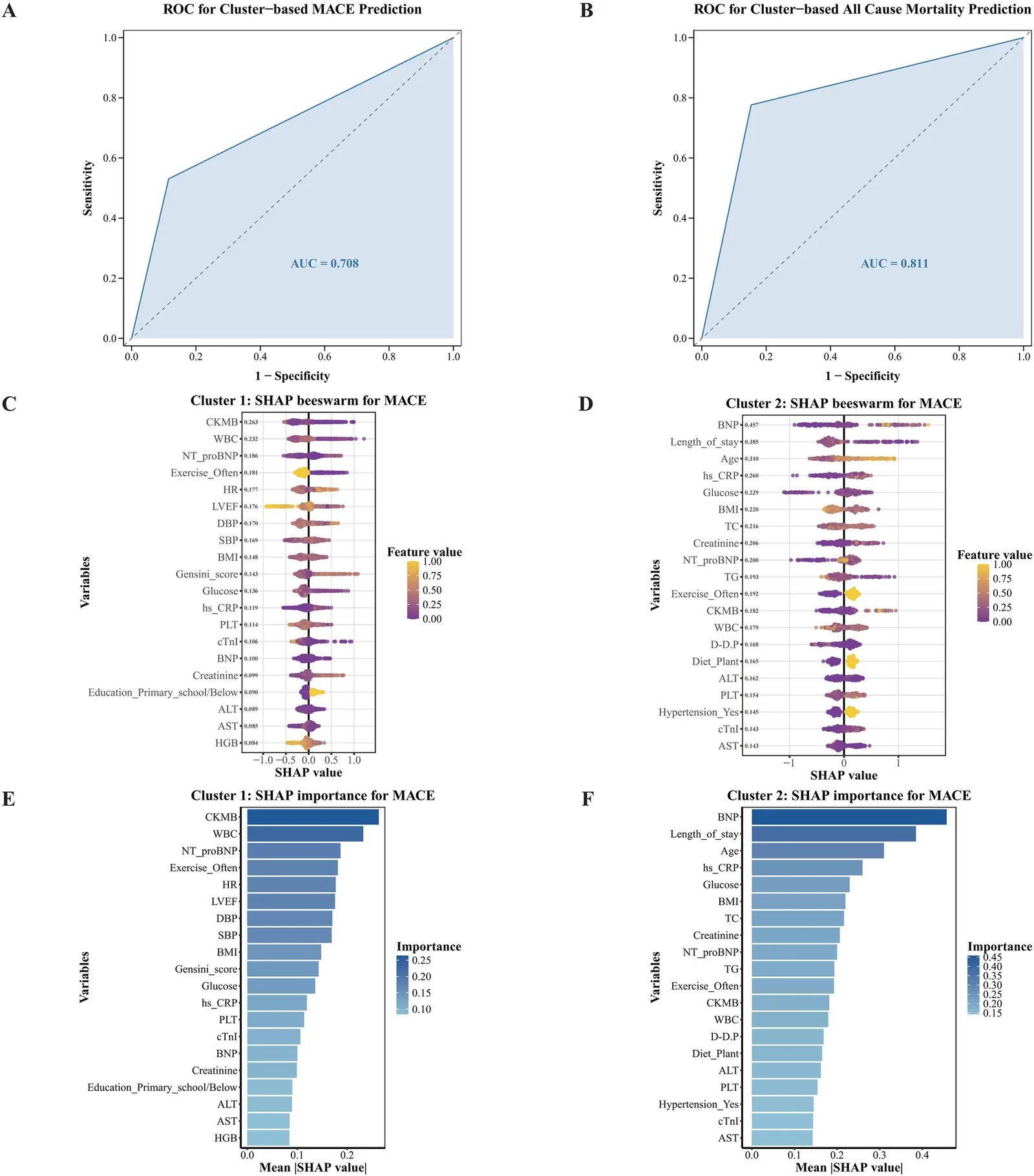
Predictive performance and variable importance of clusters. **(A)** ROC curve for cluster-based prediction of MACE. **(B)** ROC curve for cluster-based prediction of all-cause mortality. **(C–F)** SHAP analyses for cluster membership: **(C)** SHAP summary plot for Cluster 1 (MACE). **(D)** SHAP summary plot for Cluster 2 (MACE). **(E)** Mean SHAP values for Cluster 1 (MACE). **(F)** Mean SHAP values for Cluster 2 (MACE).

To further explore which variables primarily drove cluster formation, XGBoost models were developed separately within Cluster 1 and Cluster 2. SHAP values were computed to quantify variable contributions within each cluster, and the top 20 variables ranked by SHAP values were selected for visualization. The six most important variables in Cluster 1 were CK-MB, WBC, NT-proBNP, Exercise (Often), HR and LVEF (Figures 3C,E). The six most important variables in Cluster 2 were BNP, length of hospital stay, age, hs-CRP, glucose, and BMI (Figures 3D,F). Although top-ranked variables in both clusters included markers of inflammation and myocardial injury, such as WBC, CK-MB, NT-proBNP, and BNP, higher BNP values in Cluster 2 were predominantly distributed on the right side of the beeswarm plot, indicating a strong positive contribution to risk. These findings suggest that inflammation and myocardial injury may be key intrinsic drivers of the elevated risk observed in Cluster 2.

To verify whether the top-ranked variables possessed good predictive performance for distinguishing each cluster, prediction models were constructed using the top 6 variables from each cluster, with the feature set fixed before cross-validation. Model performance was evaluated using 5-fold cross-validation. The out-of-fold AUC was 0.6076 (95% CI 0.5577–0.6575) for Cluster 1 and 0.7078 (95% CI 0.6427–0.7728) for Cluster 2 (Supplementary Figure S12). The relatively higher AUC for Cluster 2 suggests that the top 6 variables captured the characteristic feature profile of this high-risk cluster more effectively, consistent with the fact that Cluster 2 represented a more homogeneous “frailty–inflammation” phenotype driven by a distinct set of dominant variables. In contrast, the lower AUC for Cluster 1 likely reflected its greater internal heterogeneity, as it comprised the majority of patients with more varied clinical profiles, making the cluster harder to separate from the rest using only the top-ranked variables.

## 4 Discussion

This work integrates multidimensional data from 1,139 patients with AMI and applies a hierarchical agglomerative clustering algorithm to characterize phenotypic heterogeneity, identifying two clinical phenotypes with differing pathophysiological characteristics. Cluster 1 (*n* = 896, 78.7%) predominantly comprises younger men and is characterized by metabolic abnormalities with preserved functional status, reflected by higher HGB, TC, TG, LDL-C, and LVEF, along with lower levels of inflammatory markers (e.g., hs-CRP and PCT) (15) and heart failure markers (cTnI, BNP, and NT-proBNP) (16). This subtype is therefore considered low risk. In contrast, Cluster 2 (*n* = 243, 21.3%) exhibits a high-risk “frailty–inflammation” phenotype, characterized by older age, a higher proportion of women, increased risk of falls and pressure ulcers, lower BMI, and elevated levels of inflammatory and heart failure markers (Supplementary Table S2). This phenotypic distinction is consistent with the “metabolic–inflammator*y* axis” proposed in cardiovascular disease (17–19), suggesting that these subtypes may be driven by distinct pathophysiological mechanisms.

Survival analysis indicates that Cluster 2 has a higher cumulative incidence of MACE at 1 year compared with Cluster 1. Risks for individual MACE components, including all-cause mortality, recurrent myocardial infarction, refractory angina, and new-onset heart failure, are also generally elevated in Cluster 2. Multivariable Cox regression analysis confirms Cluster 2 membership as an independent predictor for MACE, with a particularly strong association with recurrent myocardial infarction. From a phenotypic perspective, Cluster 2 shows abnormalities across frailty-related measures, inflammatory biomarkers, and health-related behaviors, which together define the core profile of the high-risk “frailty–inflammation” phenotype. Stratified analyses further demonstrated that the prognostic association of this phenotype was directionally consistent across STEMI and NSTEMI, with no significant interaction, suggesting that the identified phenotype and its adverse prognostic implications are applicable to both infarction types (Supplementary Figure S11; Table S15).

The principal innovation lies in addressing a key limitation of previous phenotyping studies in AMI. Conventional approaches predominantly focus on baseline clinical data, laboratory parameters, and procedural variables (20–22). In contrast, this work integrates clinical features, metabolic characteristics, and behavioral patterns into a machine learning–based clustering framework, thereby incorporating multidimensional patient data. This approach provides a more comprehensive basis for identifying high-risk phenotypes. Previous stratification studies in AMI largely rely on traditional statistical methods or limited machine learning approaches, with variables typically restricted to age, comorbidities, cardiac function indices, and biochemical markers (23, 24). These approaches often overlook the contribution of frailty and health-related behaviors to long-term outcomes. Existing evidence indicates that frailty reduces physiological reserve and increases the risk of post-procedural complications in patients with AMI (25, 26), while chronic inflammation contributes to endothelial dysfunction and accelerates atherosclerosis progression (27). The coexistence of these processes may establish a self-perpetuating “frailty–inflammation cycle” (28). Integration of multidimensional data enables clear delineation of this high-risk phenotype and its strong association with MACE. This work therefore provides a novel biological–functional–behavioral framework for risk stratification in AMI.

The elevated incidence of MACE in Cluster 2 may be associated with the synergistic interaction of a “frailty–inflammation–poor health behavior” triad. The following three pathways are proposed as potential mechanisms based on prior biological evidence; they remain speculative in the context of the present data and require confirmation through prospective and mechanistic studies. First, physical frailty and functional decline: Patients in Cluster 2 exhibit low MBI scores and increased risks of falls and pressure ulcers, indicating functional decline that may reflect reduced muscle mass and diminished metabolic and stress response capacity. This functional impairment may limit myocardial repair after AMI and increase susceptibility to complications, such as thrombosis and pulmonary infections related to reduced mobility, potentially contributing to elevated MACE risk (29). Second, a sustained pro-inflammatory state: This subtype is characterized by elevated hs-CRP and PCT levels, indicating persistent systemic inflammation. Inflammatory cytokines may activate the coagulation system, disrupt endothelial function, and accelerate plaque progression or rupture, thereby potentially contributing to an increased risk of recurrent myocardial infarction and stroke (30). Third, adverse health behaviors: Patients in Cluster 2 show insufficient sleep duration and suboptimal dietary patterns, along with markedly low levels of physical activity. Although a mechanistic link between these behavioral factors and recurrent cardiovascular events is biologically plausible through their effects on systemic inflammation and autonomic function, the present observational data cannot establish whether these behaviors play a causal role in the observed outcome differences. These behaviors may exacerbate the inflammatory state and reduce the effectiveness of pharmacological therapies, potentially amplifying the adverse prognostic impact of the frailty–inflammation cycle. Prospective and mechanistic studies are warranted to further substantiate these proposed pathways.

Despite the strengths discussed above, several limitations should be considered. First, the observational design introduces the possibility of regional and population selection bias, which may limit generalizability to other geographic or ethnic populations. Second, the 1-year follow-up period is insufficient to assess long-term prognostic impact. Third, although internal validation was performed, cluster stability was moderate: clusterboot Jaccard coefficients were 0.623 and 0.753 for Cluster 1 and Cluster 2, respectively; the median adjusted Rand index across 80% subsamples was 0.19; the mean silhouette width for Cluster 2 was −0.097; and permutation testing indicated that the cluster structure was sensitive to the overall variable list (Supplementary Figures S1, 4 and 6). These findings indicate that the identified phenotypic structure is not geometrically perfect and should be interpreted with appropriate caution. Nevertheless, the pronounced difference in clinical outcomes between clusters—approximately 3.2-fold for MACE (Cluster 1 vs. 2 = 14.4% vs. 46.5%) and 9.7-fold for all-cause mortality (Cluster 1 vs. 2 = 2.5% vs. 24.3%) —remains the core evidence supporting the clinical relevance of the two-cluster solution, complementing the moderate stability metrics. Fourth, the lack of external validation means that the robustness and generalizability of the phenotypic model require confirmation in multi-center datasets. External validation in multi-center cohorts or public databases (e.g., the MIMIC-IV AMI subgroup) is planned, with evaluation of the adjusted Rand index, silhouette coefficients, and between-cluster outcome HRs. Fifth, the all-cause mortality analysis had an EPV of approximately 4.5, which is below the conventional threshold, and calibration analyses indicated a tendency toward overestimation of absolute risk in high-risk subgroups. Finally, although an association between the “frailty–inflammation” phenotype and MACE is identified, the underlying molecular mechanisms are not explored. Further studies incorporating experimental approaches are required to clarify these mechanisms.

In conclusion, these findings extend current approaches to AMI phenotyping. In contrast to conventional classifications based on anatomical features or biomarkers, this work integrates multidimensional data, including metabolic, inflammatory, physical, and behavioral factors, to identify a high-risk subgroup not captured by traditional systems. These results may support a continuum of AMI care, from risk identification to precision intervention, and may offer practical value, particularly for high-risk populations that may be under-recognized by conventional phenotyping approaches. However, these clinical implications are based on observational associations and should be interpreted with caution until validated in independent cohorts. The proposed phenotype, while promising, does not yet warrant direct application in routine clinical practice; multi-center prospective studies are required to confirm its robustness and generalizability.

### 4.1 Implications and future directions

Beyond its implications for risk stratification, the “frailty–inflammation” phenotype identified in this study may also inform clinical and nursing practice, suggesting several potential directions for future translation: First, electronic medical record–based risk flagging and early warning: patients classified as the high-risk phenotype could be automatically labeled at admission, triggering high-risk monitoring protocols (31). The effectiveness of such automated alert systems, however, requires prospective evaluation. Second, stratified nursing interventions: for Cluster 2 patients, care priorities would emphasize frailty management (nutritional support, fall and pressure ulcer prevention), infection prevention, and heart failure management; for Cluster 1 patients, efforts would focus on reinforcing lifestyle interventions and secondary prevention medication adherence (32). Third, optimized allocation of nursing resources: limited intensive monitoring resources could be prioritized for high-risk phenotype patients, potentially reducing readmission rates in this subgroup (33). It should be emphasized that none of these strategies was assessed in the present study; they represent directions for future research and application, and should not enter clinical practice until confirmed by independent validation and prospective interventional studies.

## 5 Conclusions

Integration of multidimensional data from patients with AMI using hierarchical agglomerative clustering identified two clinical phenotypes with differing pathophysiological characteristics. Cluster 2, comprising 21.3% of the cohort, represents a high-risk “frailty–inflammation” phenotype that is associated with increased risks of 1-year MACE and all-cause mortality, and was identified as an independent predictor of poor prognosis after AMI. By incorporating clinical, metabolic, functional, and behavioral dimensions into a unified analytical framework, this approach addresses limitations of previous phenotyping studies and provides a more comprehensive basis for early identification of high-risk patients, potentially informing the development of targeted intervention strategies. However, these findings, while promising, do not yet support the routine clinical application of this phenotype until validated in independent cohorts. Future research should prioritize: (1) external validation of phenotypic stability and prognostic associations in multi-center cohorts or public databases (e.g., the MIMIC-IV AMI subgroup); (2) prospective evaluation of whether phenotype-guided stratified interventions can improve outcomes; and (3) mechanistic investigation of the biological pathways underlying the “frailty–inflammation” axis.

## Data Availability

The datasets generated and/or analyzed during the current study are not publicly available due to privacy and ethical restrictions but are available from the corresponding author upon reasonable request.
