# Supplementary material for: Phenotyping of AMI patients using machine learning: integration of clinical, metabolic, and behavioral features to identify high-risk “frailty–inflammation” phenotype
Source: Front Cardiovasc Med. 2026 Oct 9;13:1881280. doi: 10.3389/fcvm.2026.1881280 (PMC13690802; doi:10.3389/fcvm.2026.1881280)
Supplement: Supplementary file 1 [file Datasheet1.pdf]

Supplementary Figures

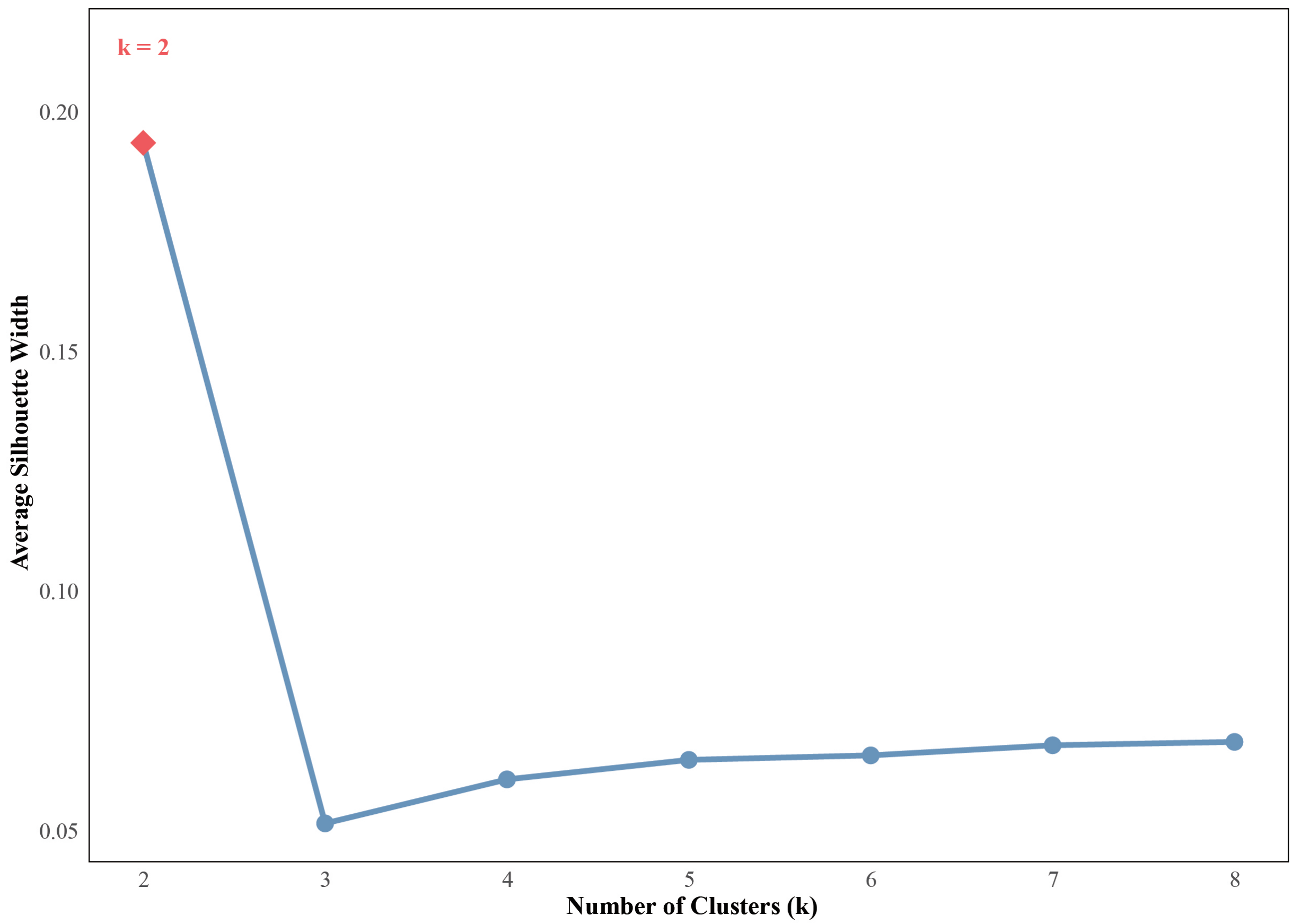

Figure S1. Silhouette method for optimal clusters. The clustering performance was evaluated using the average silhouette width across different numbers of clusters (k = 2 – 8). The average silhouette width was highest at k = 2 (approximately 0.19).

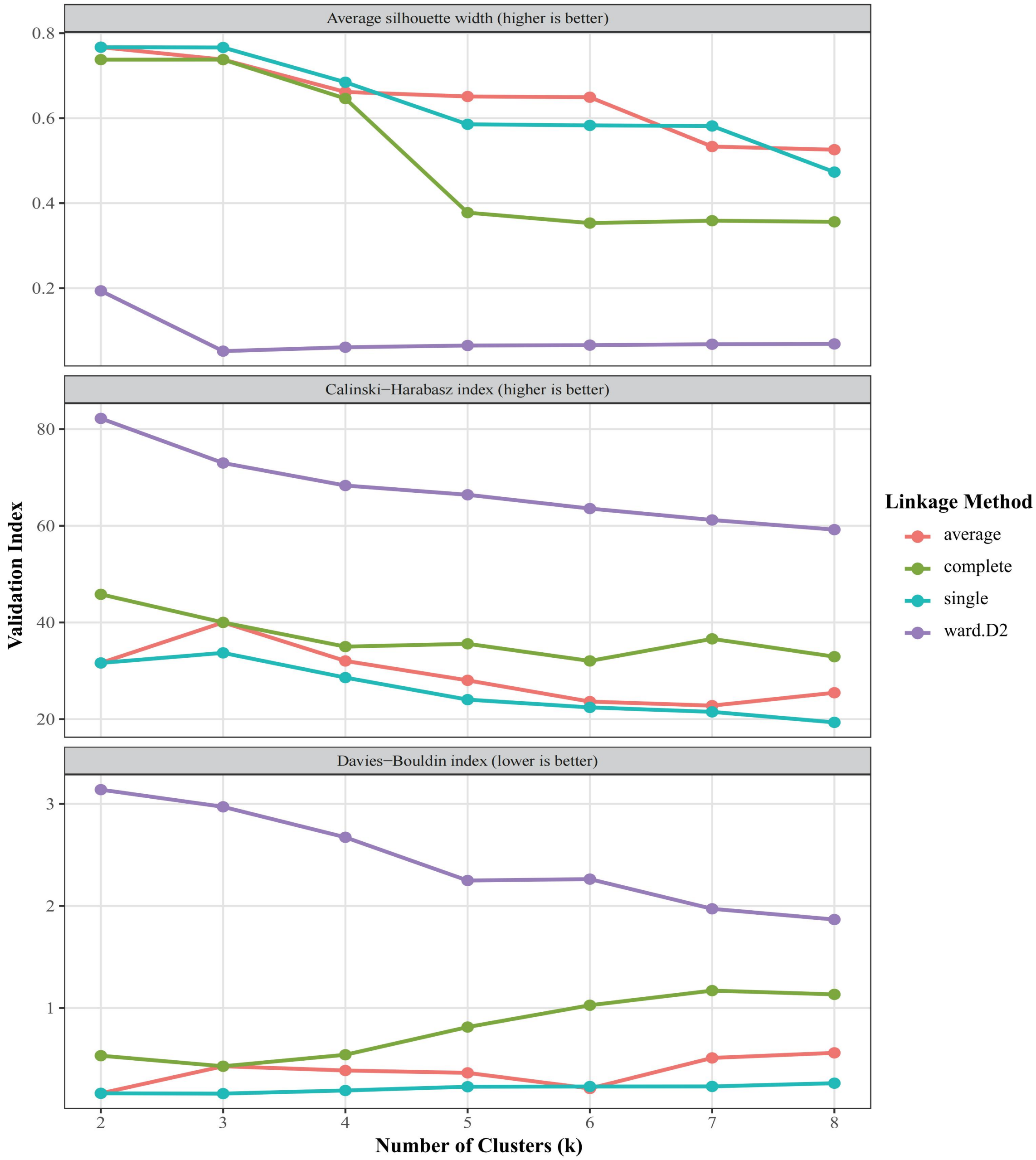

Figure S2. Internal validation across linkage methods. Clustering performance was evaluated using the average silhouette width, Calinski–Harabasz index, and Davies–Bouldin index for k = 2–8 with four linkage methods: average, complete, single, and Ward.D2. Higher values indicate better clustering for the average silhouette width and Calinski–Harabasz index, whereas lower values indicate better clustering for the Davies–Bouldin index.

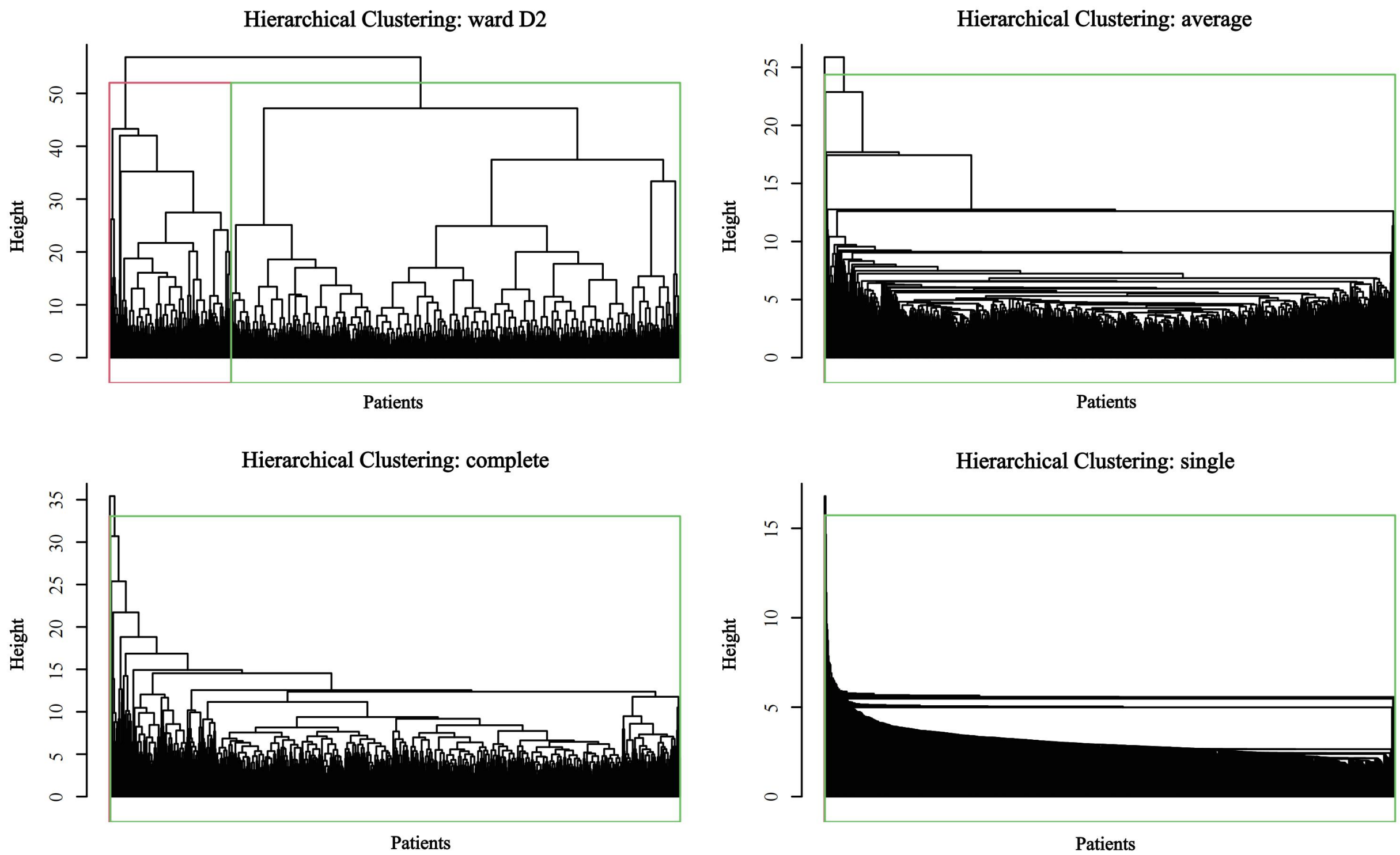

Figure S3. Dendrogram comparison by linkage method. Hierarchical clustering of patients was performed using the Ward.D2, average, complete, and single linkage methods. The dendrograms illustrate the clustering structure and merge heights obtained with each method.

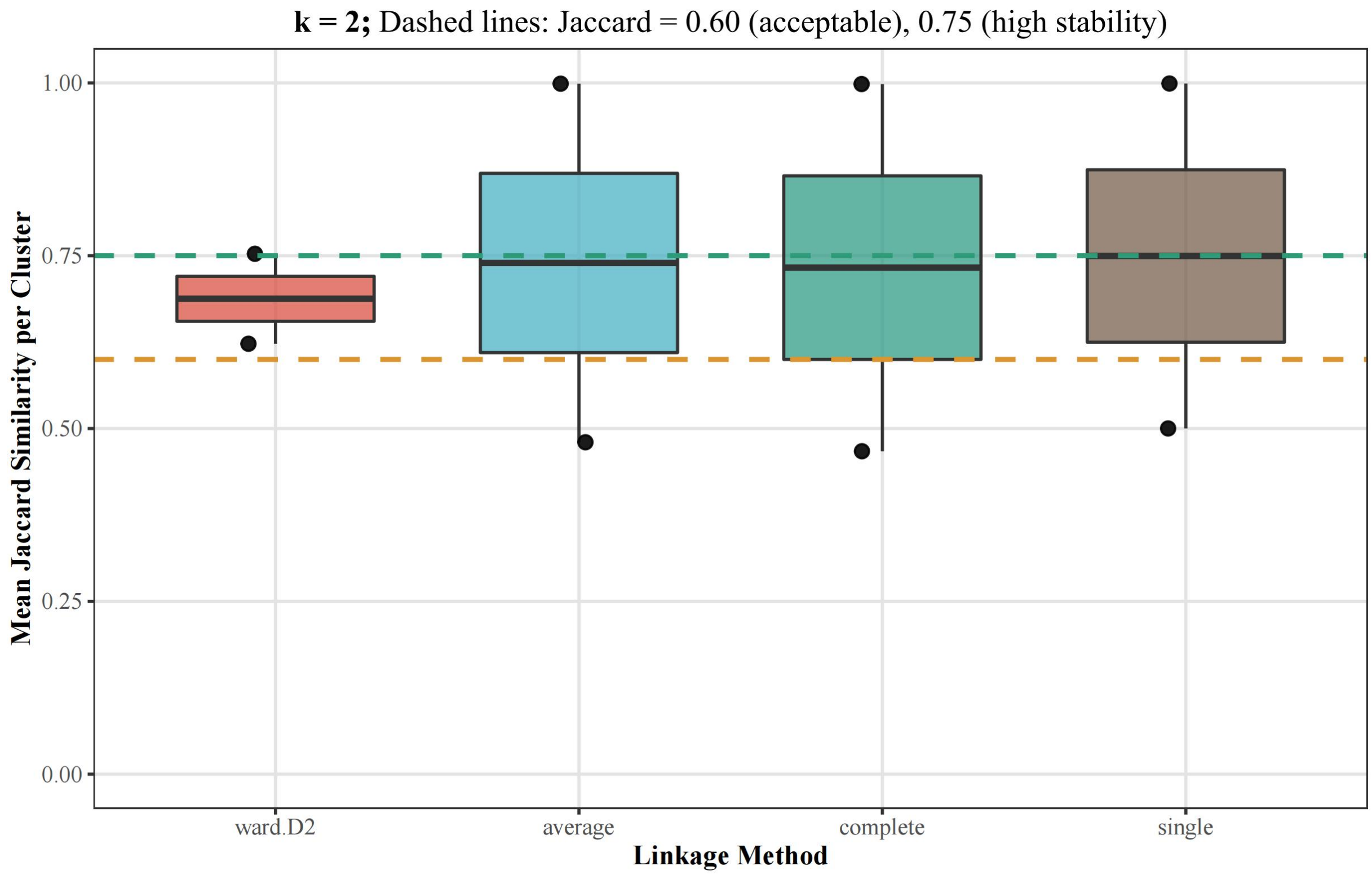

Figure S4. Bootstrap cluster stability comparison. For k = 2, clustering stability was evaluated using the mean Jaccard similarity per cluster for the Ward.D2, average, complete, and single linkage methods. Dashed lines indicate Jaccard similarity values of 0.60 (acceptable stability) and 0.75 (high stability).

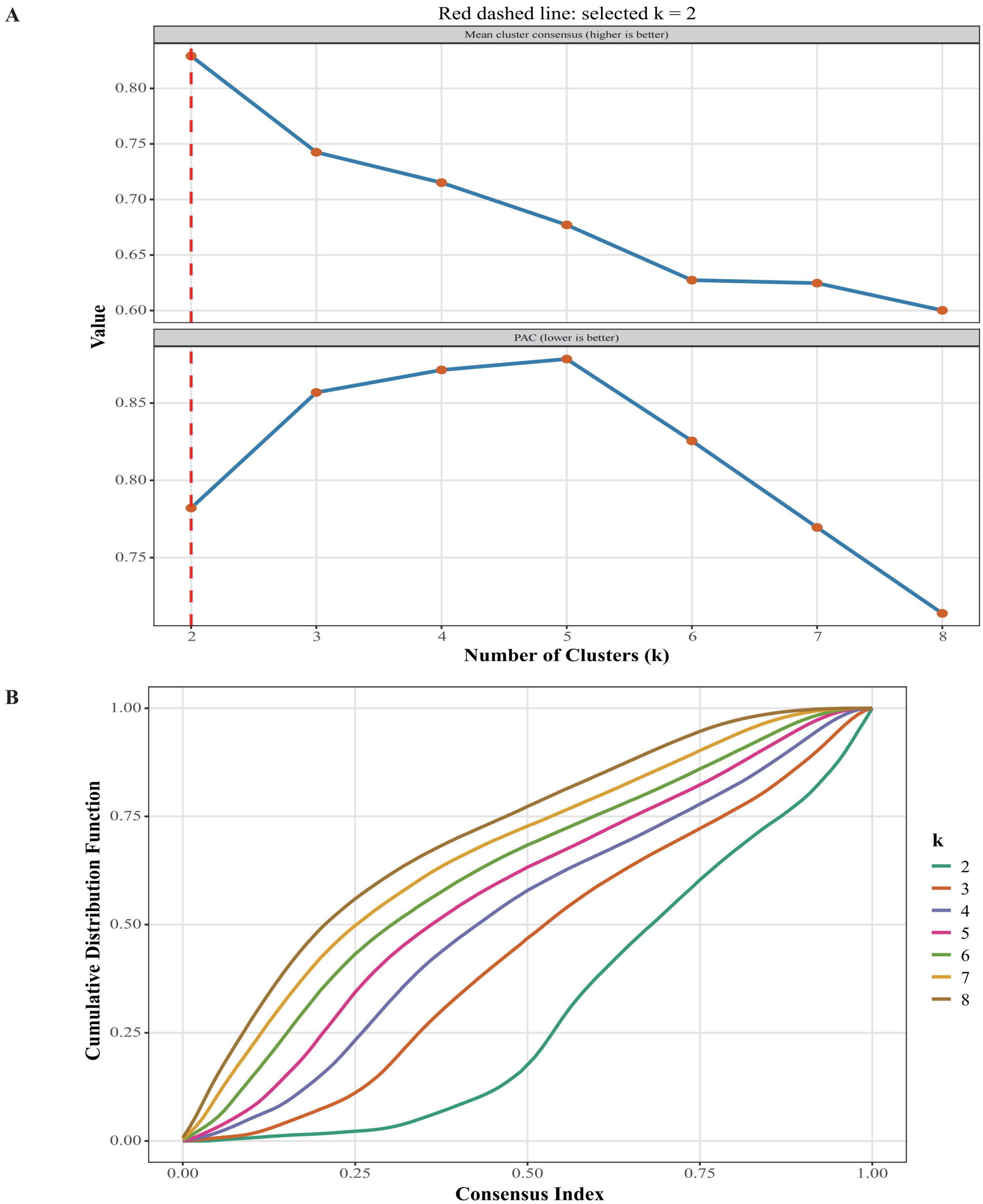

Figure S5. Consensus clustering analysis. (A) Mean cluster consensus and PAC values were compared across different numbers of clusters (k = 2−8), with the red dashed line indicating the selected solution of k = 2. (B) Cumulative distribution functions of the consensus index for different numbers of clusters. PAC, proportion of ambiguous clustering.

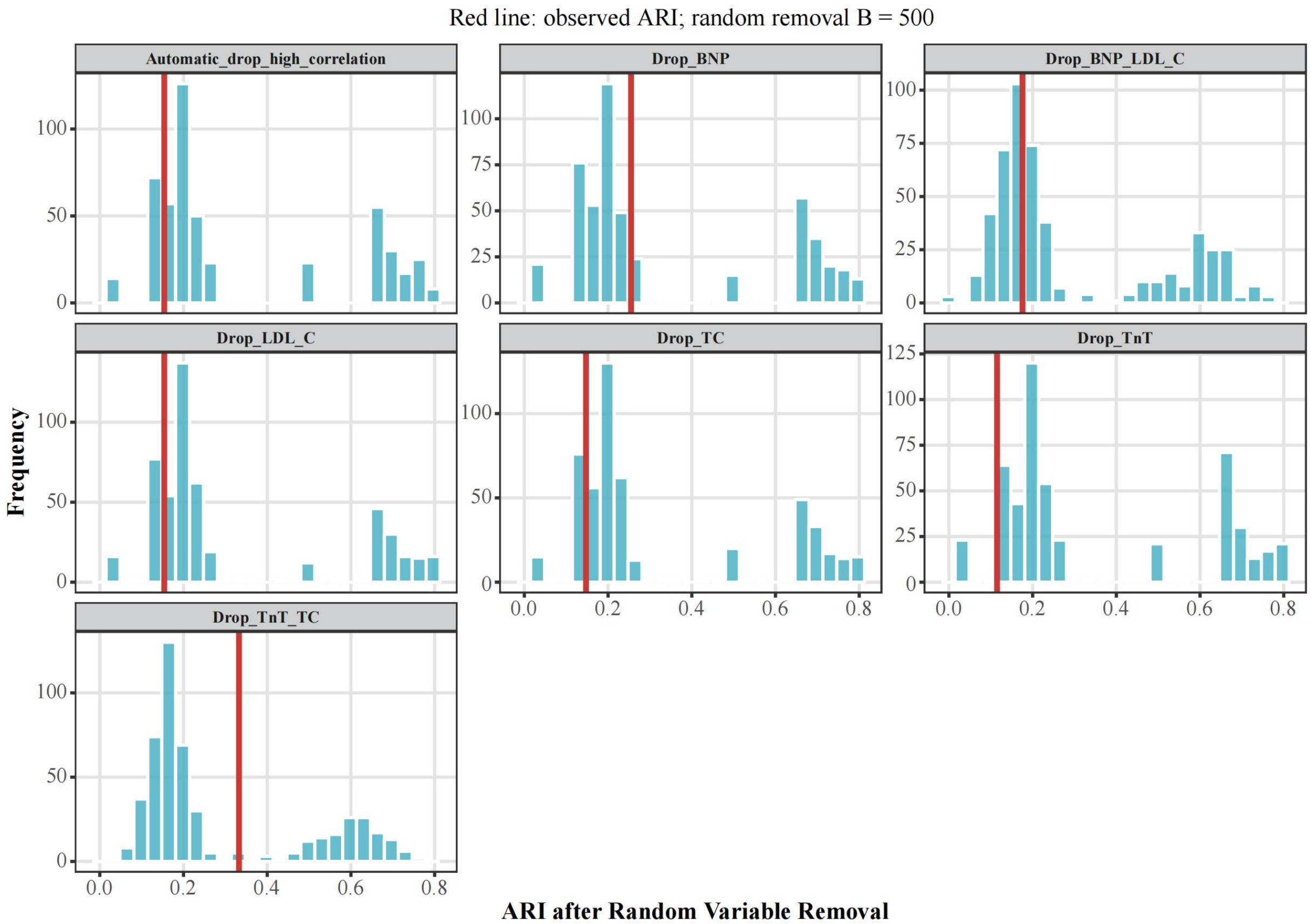

Figure S6. Random equal-size variable removal distribution. To determine whether the clustering structure was primarily driven by a limited number of variables, equal numbers of variables were randomly removed and the procedure was repeated for B = 500 permutations under each variable-removal scheme to generate a null distribution of the adjusted Rand index (ARI). Blue histograms show the null distributions of ARI after random variable removal, whereas red vertical lines indicate the observed ARI in the original data.

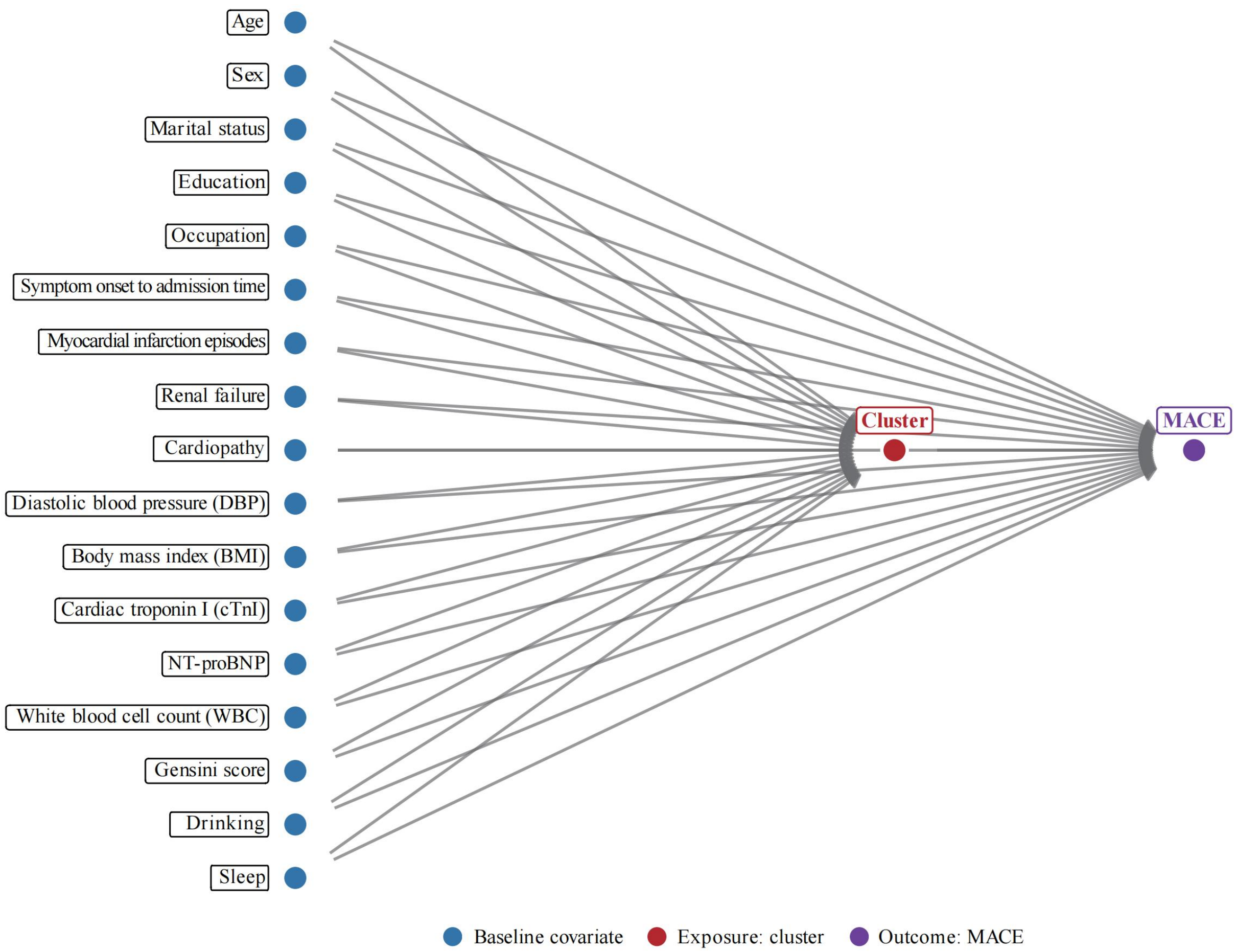

Figure S7. Prespecified causal DAG for cluster and MACE. Baseline covariates included age, sex, marital status, education, occupation, symptom onset to admission time, myocardial infarction episodes, renal failure, cardiopathy, diastolic blood pressure (DBP), body mass index (BMI), cardiac troponin I (cTnI), NT-proBNP, white blood cell count (WBC), Gensini score, drinking, and sleep; cluster was treated as the exposure and MACE as the outcome. DAG, directed acyclic graph.

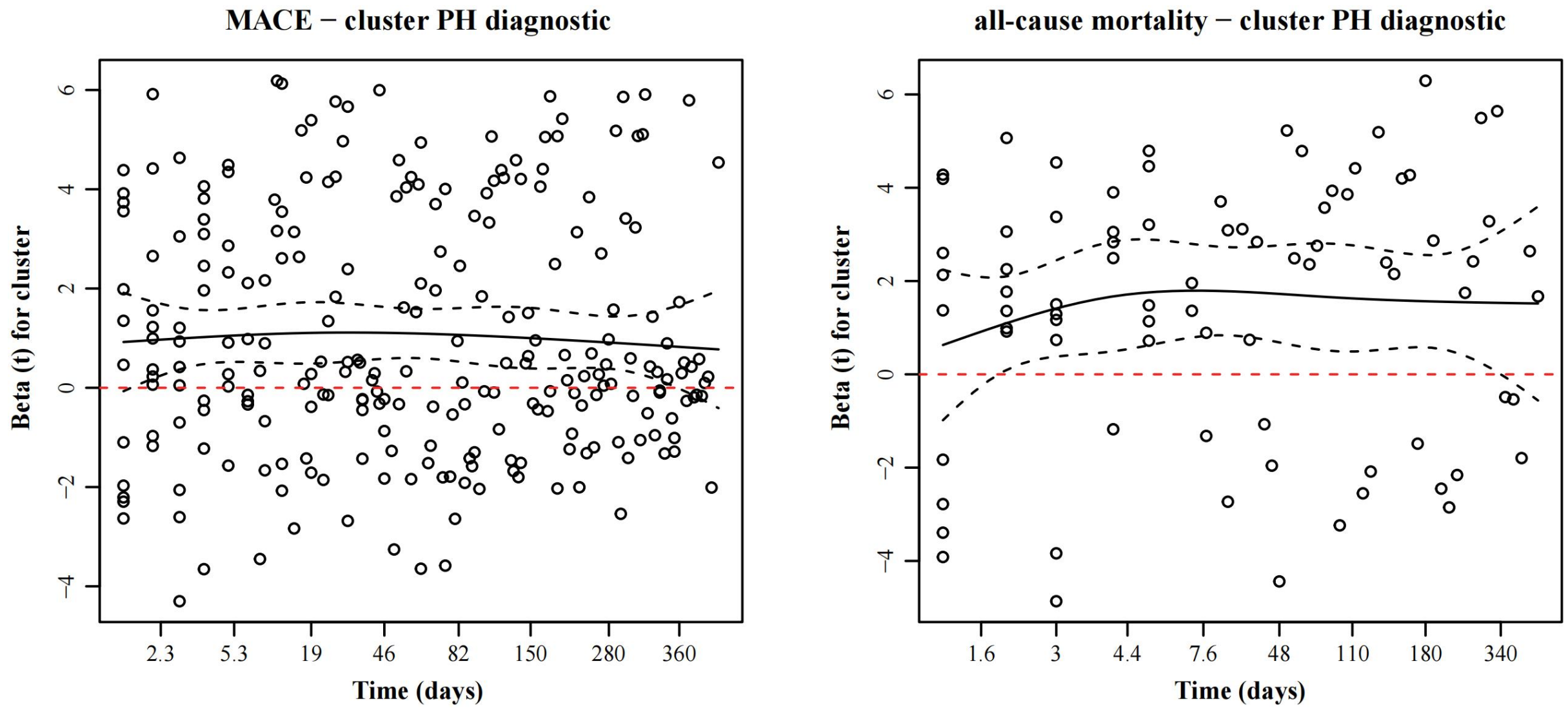

Figure S8. MACE and all-cause mortality M3 cluster Schoenfeld plot. The proportional hazards assumption for the association of cluster membership with MACE and all-cause mortality was assessed using time-varying regression coefficients and their 95% confidence intervals. The solid lines represent the estimated time-varying coefficients, the dashed lines indicate the 95% confidence intervals, and the red horizontal line denotes a coefficient of zero.

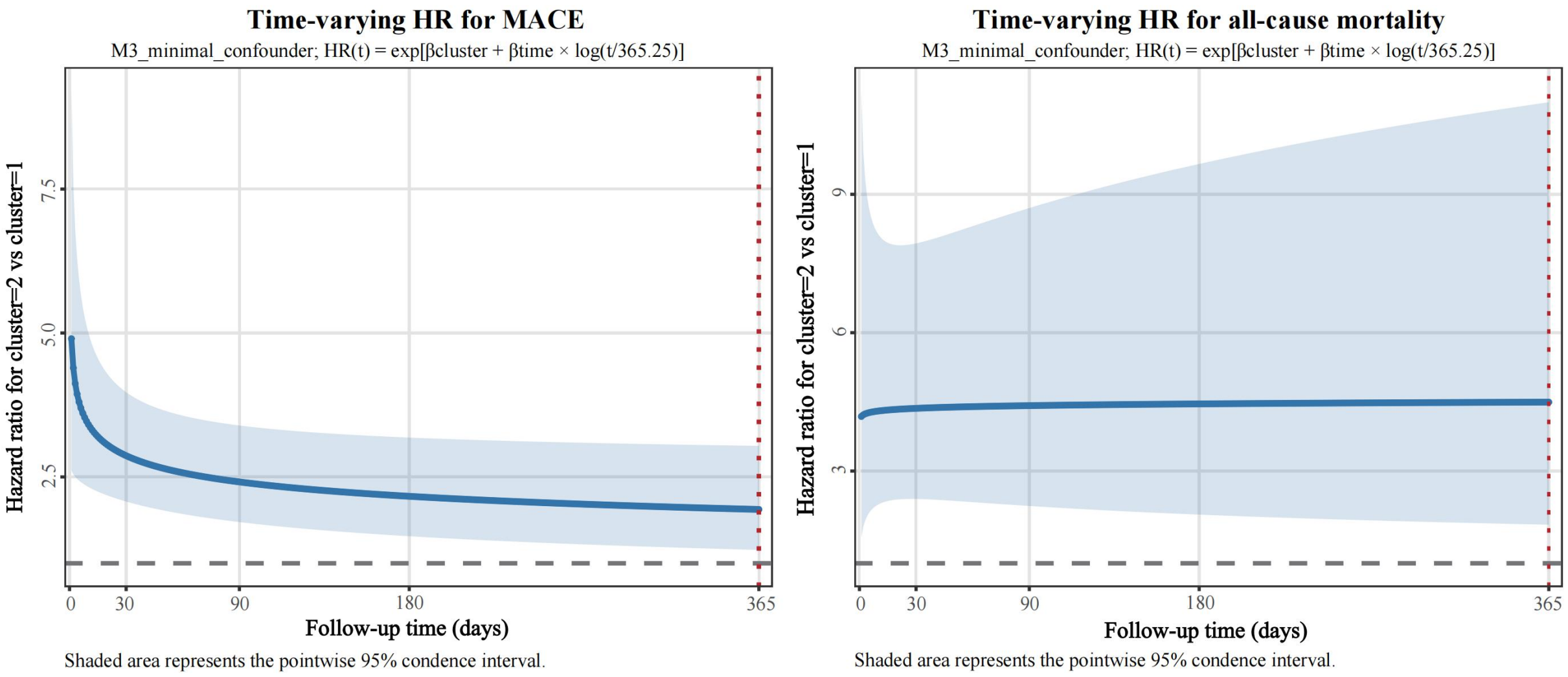

Figure S9. Combined time-varying HR plots. Cox regression models with a time interaction term were used to estimate the HR for cluster 2 versus cluster 1 over 365 days of follow-up for MACE and all-cause mortality. The blue solid line represents the estimated time-varying HR, the shaded area indicates the 95% confidence interval, and the gray dashed line denotes HR = 1. HR, hazard ratio.

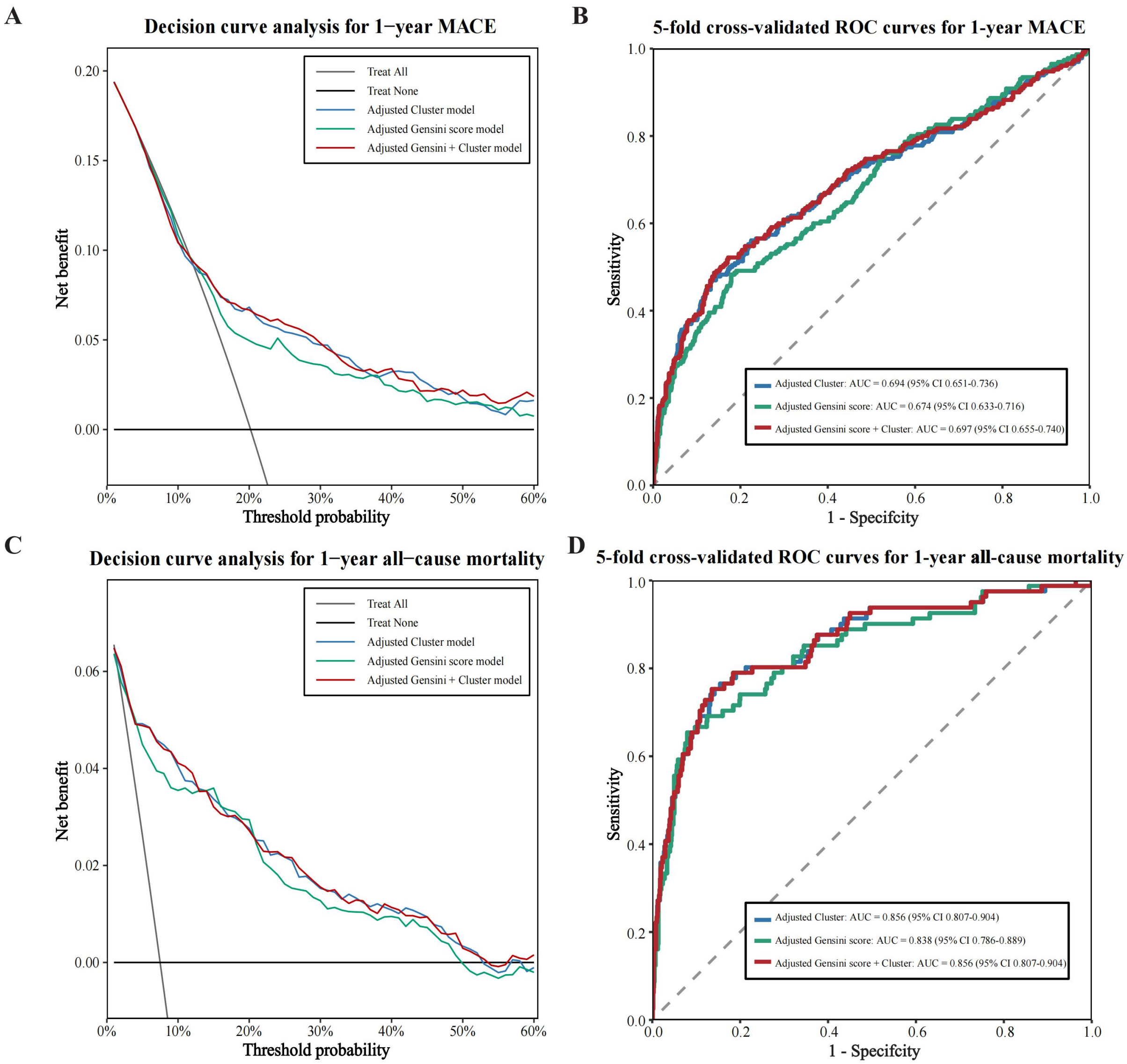

Figure S10. Decision curve analysis and 5-fold cross-validated ROC curves for 1-year MACE and all-cause mortality. Panels A and C show decision curve analyses for 1-year MACE and all-cause mortality, respectively, comparing the net benefit of the adjusted cluster model, adjusted Gensini score model, and combined adjusted Gensini score plus cluster model across threshold probabilities. The gray diagonal line represents the strategy of assuming that no patients experience the outcome, whereas the black curve represents the strategy of assuming that all patients experience the outcome. Panels B and D show the 5-fold cross-validated ROC curves for 1-year MACE and all-cause mortality, respectively, comparing the discriminative performance of the models. The area under the curve (AUC) with its 95% confidence interval is provided in the legends, and the gray dashed diagonal line indicates the reference for no discrimination (AUC = 0.50).

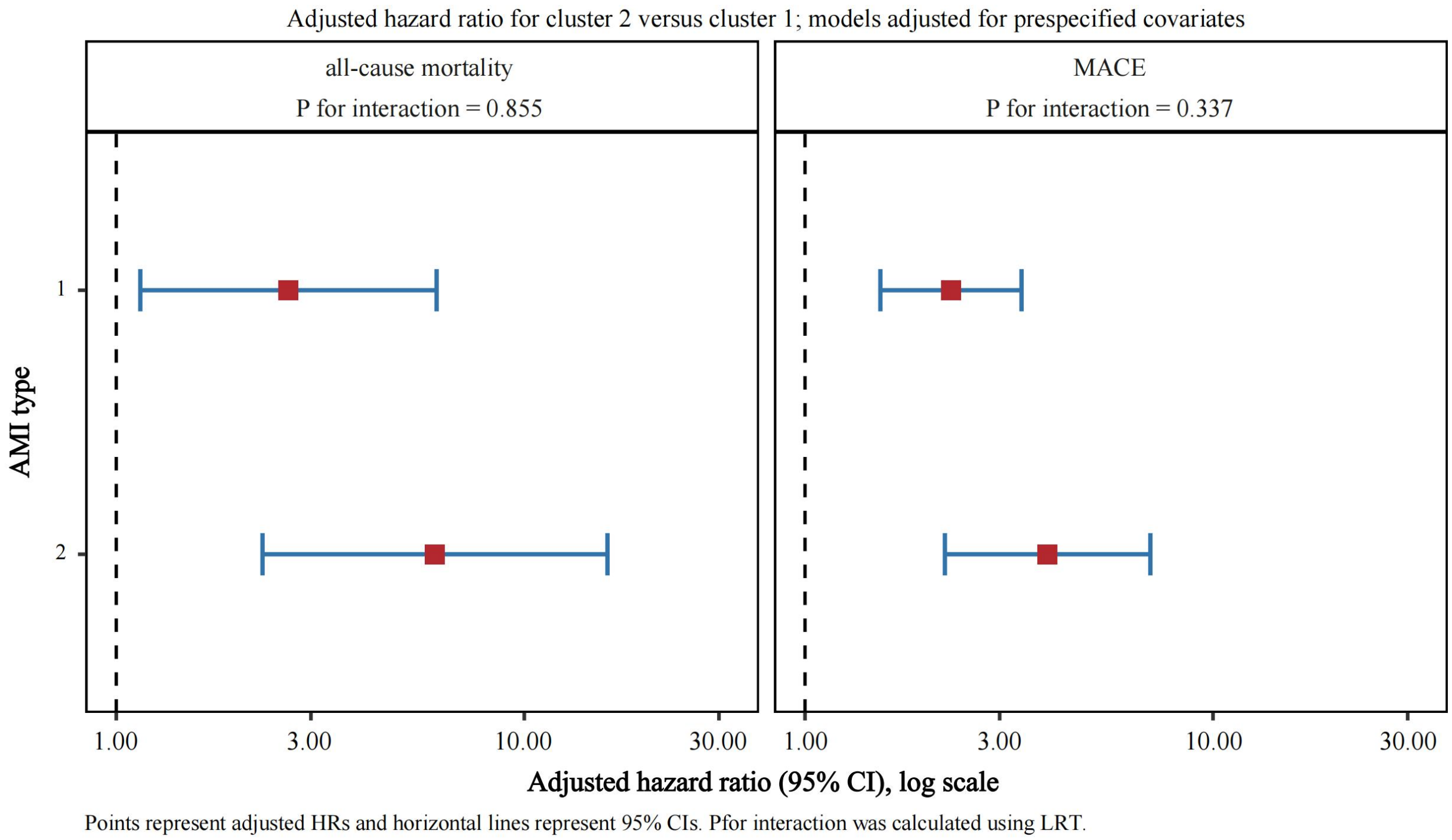

Figure S11. AMI type stratified cluster HR forest plot. The forest plot shows adjusted hazard ratios (HRs) and 95% confidence intervals for cluster 2 versus cluster 1 in relation to all-cause mortality and major adverse cardiovascular events (MACE), stratified by AMI type: type 1 AMI, defined as STEMI, and type 2 AMI, defined as NSTEMI. Red squares represent the adjusted HRs, horizontal lines indicate the 95% confidence intervals, and the dashed line denotes HR = 1. P values for interaction assess whether AMI type modifies the associations between cluster membership and the outcomes. STEMI, ST-segment elevation myocardial infarction; NSTEMI, non-ST-segment elevation myocardial infarction.

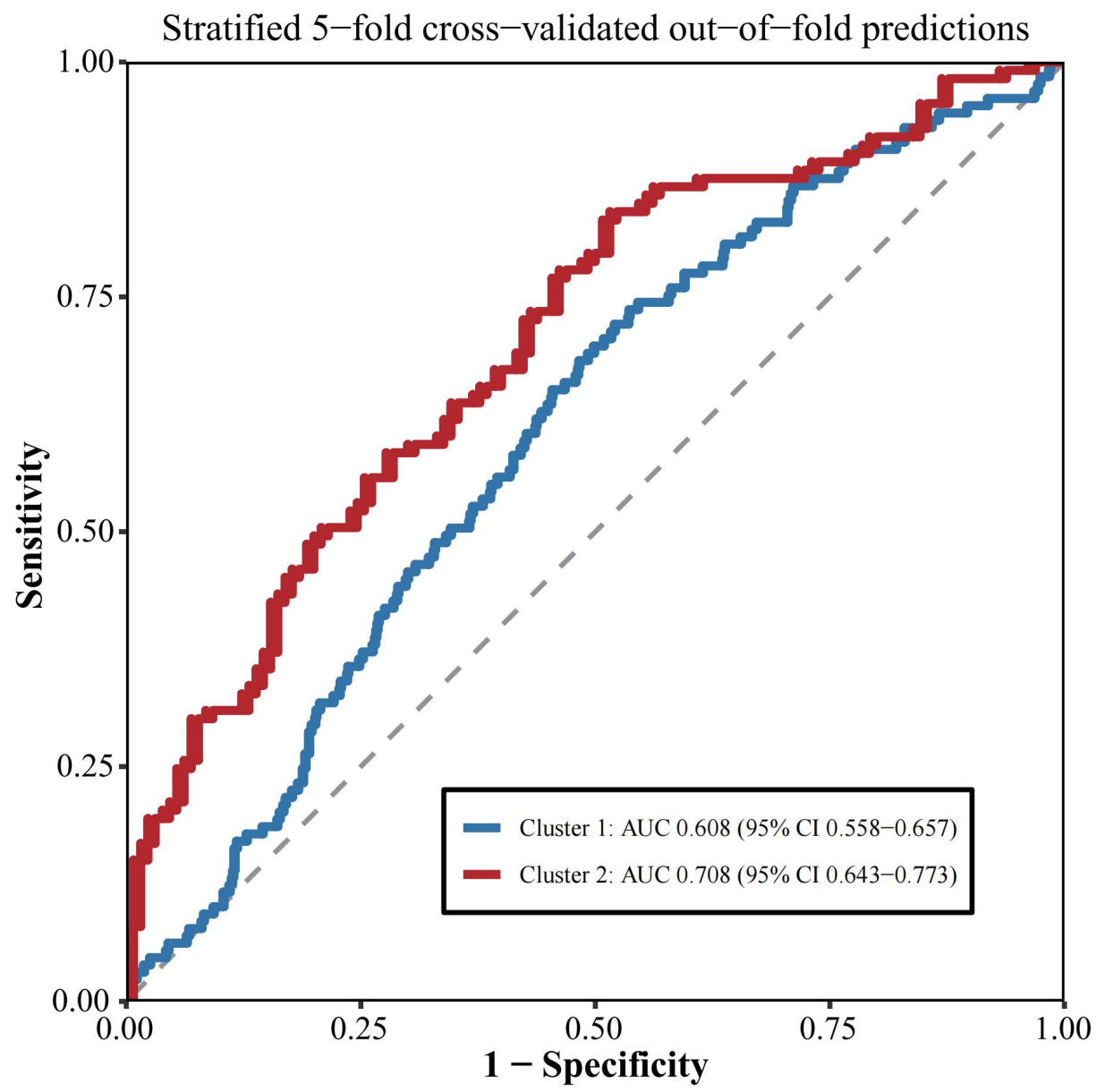

Figure S12. ROC curve based on fixed SHAP top 6 features. The ROC curves evaluated the ability of the top six most important variables identified for clusters 1 and 2 to predict membership in their respective clusters. The area under the curve (AUC) was 0.608 (95% CI, 0.558 – 0.657) for cluster 1 and 0.708 (95% CI, 0.663 – 0.773) for cluster 2. The gray dashed diagonal line represents no discrimination (AUC = 0.50).
